# Extent and types of gender-based discrimination against female medical students and physicians at five university hospitals in Germany – results of an online survey

**DOI:** 10.3205/zma001648

**Published:** 2023-11-15

**Authors:** Jan-Filip Tameling, Mareike Lohöfener, Judith Bereznai, Thi Phuong Anh Tran, Marie Ritter, Margarete Boos

**Affiliations:** 1 Georg-August University Göttingen, Georg Elias Müller Institute for Psychology, Department of Social and Communication Psychology, Göttingen, Germany

**Keywords:** gender-based discrimination, qualitative analysis, female students, female physicians

## Abstract

**Objective:**

There is a gap in research on gender-based discrimination (GBD) in medical education and practice in Germany. This study therefore examines the extent and forms of GBD among female medical students and physicians in Germany. Causes, consequences and possible interventions of GBD are discussed.

**Methods:**

Female medical students (*n*=235) and female physicians (*n*=157) from five university hospitals in northern Germany were asked about their personal experiences with GBD in an online survey on self-efficacy expectations and individual perceptions of the “glass ceiling effect” using an open-ended question regarding their own experiences with GBD. The answers were analyzed by content analysis using inductive category formation and relative category frequencies.

**Results:**

From both interviewed groups, approximately 75% each reported having experienced GBD. Their experiences fell into five main categories: sexual harassment with subcategories of verbal and physical, discrimination based on existing/possible motherhood with subcategories of structural and verbal, direct preference for men, direct neglect of women, and derogatory treatment based on gender.

**Conclusion:**

The study contributes to filling the aforementioned research gap. At the hospitals studied, GBD is a common phenomenon among both female medical students and physicians, manifesting itself in multiple forms. Transferability of the results beyond the hospitals studied to all of Germany seems plausible. Much is known about the causes, consequences and effective countermeasures against GBD. Those responsible for training and employers in hospitals should fulfill their responsibility by implementing measures from the set of empirically evaluated interventions.

## Zusammenfassung

**Zielsetzung:**

Zur genderbasierten Diskriminierung (GBD) in der medizinischen Ausbildung und Tätigkeit besteht in Deutschland eine Forschungslücke. Die vorliegende Studie untersucht daher Ausmaß und Formen von GBD bei Medizinstudentinnen und Ärztinnen in Deutschland. Es werden Ursachen, Folgen und Interventionsmöglichkeiten von GBD diskutiert.

**Methodik:**

Medizinstudentinnen (*n*=235) und Ärztinnen (*n*=157) von fünf Universitätskrankenhäusern in Norddeutschland wurden im Rahmen einer Online-Umfrage zu Selbstwirksamkeitserwartung und Glasdeckeneffekt anhand einer offenen Frage nach ihren persönlichen Erfahrungen mit GBD befragt. Die Antworten wurden inhaltsanalytisch mittels induktiver Kategorienbildung und nach relativen Kategorienhäufigkeiten ausgewertet.

**Ergebnisse:**

Aus beiden befragten Gruppen gaben jeweils ca. 75% an, GBD erlebt zu haben. Ihre Erfahrungen lassen sich in fünf Hauptkategorien unterteilen: sexuelle Belästigung mit den Unterkategorien verbal und körperlich, Diskriminierung aufgrund bestehender/möglicher Mutterschaft mit den Unterkategorien strukturell und verbal, unmittelbare Bevorzugung von Männern, unmittelbare Vernachlässigung von Frauen und herabwürdigender Umgang aufgrund des Geschlechts.

**Schlussfolgerung:**

Die Studie leistet einen Beitrag, um die genannte Forschungslücke zu schließen. An den untersuchten Krankenhäusern ist GBD sowohl bei Medizinstudentinnen als auch bei Ärztinnen ein verbreitetes Phänomen, das sich in vielfältigen Formen manifestiert. Eine Übertragbarkeit der Ergebnisse über die untersuchten Krankenhäuser hinaus auf ganz Deutschland erscheint plausibel. Über die Ursachen, Folgen und wirkungsvolle Gegenmaßnahmen gegen GBD ist viel bekannt. Verantwortliche in Ausbildung und Arbeitgeber*innen in Krankenhäusern sollten ihrer Verantwortung gerecht werden, indem sie Maßnahmen aus der Menge der empirisch evaluierten Interventionen umsetzen.

## Introduction

Research on negative experiences of students and physicians relies on constructs defined at varying levels of focus, from general *inappropriate treatment* [1] to more narrowly defined *sexual harassment* [2]. This work examines *gender-based discrimination* (GBD) and thus chooses a medium focus. GBD is defined as systematically different treatment, prejudicial attitudes, and expectations of gendered behavior of people based on their perceived membership of a gender [3], [4]. 

GBD in the educational or workplace setting is associated with negative consequences for mental health (depression, stress) [5], [6], and work-related behaviors (absenteeism, lower career aspirations) [6], [7], [8], among others. This results in economic and academic harm [6], [8]. The causes of GBD can be described at macro, meso, and micro levels [9]. At the macro level, cultural notions of acceptable behavior affect human experience and behavior. In the context of GBD, this is referred to as gender ideology (stereotypical ideas shaped by essentialist attributions) [10] and patriarchal structures (dominant and preferred position of men in society) [11], [12]. Educational and working life represents a highly pre-structured social context in which explicit and implicit organizational structures have an influence at the meso-level [13]. Regularities, such as policies, can be either inherently discriminatory (e.g., meetings at family-unfriendly times) or discriminatory in their application (e.g., not enforcing sexual harassment regulations) [14]. In addition, the implicit norms of organizational culture support or hinder GBD [15], for example, by favoring men in leadership positions in medicine [16]. A distinctive feature of organizational structures in medicine is the steep hierarchy [6]. Unequal distribution of power makes powerful individuals more likely to use stereotypes without being questioned [15], which favors the occurrence of GBD. At the micro level, individual characteristics and beliefs interact with the social context [15] to influence human behavior, including perceptual and judgment biases that operate implicitly [16]. 

GBD is a long-known [17], [18], extremely common phenomenon in medical education and work, and well documented in many countries [19], [20], [21], [22]. It particularly affects women [2], [23]. Nevertheless, GBD is sometimes considered a less relevant topic by those responsible for medical education [24], and gender disparities in medicine persist worldwide [25]. GBD can be understood as a universal phenomenon, but it can manifest itself in different behaviors depending on the culture and is also defined differently by those affected [26]. Cross-cultural comparability of results is further complicated by divergent methodological approaches [26]. It therefore seems useful to look at GBD specifically in the German-speaking world. Here it can be seen that, depending on the study, up to 58.9% of the medical students surveyed had experienced sexual harassment [27], see also [28], [29], [30], [31]. Regarding the situation of female physicians, it was reported from a hospital in northern Germany that 76% had experienced sexual harassment in some form [32]. Overall, however, the extent of sexual harassment in medical work and training in Germany has not been sufficiently studied [28], [32].

In order to contribute to closing this research gap, the present study aims firstly to investigate the extent of GBD in medical education and practice in Germany. Previous studies have focused on more narrowly defined sexual harassment. This, as systematic differential treatment based on perceived gender affiliation, represents only one of the possible forms of GBD. Accordingly, the broader focus of this work includes non-sexual differential treatment and prejudicial attitudes and expectations of gendered behavior, as included in the full definition of GBD given above, and therefore promises novel insights. Second, GBD will be studied in female medical students and physicians. To our knowledge, there is no study to date that surveys the GBD experiences of these groups of individuals together, making them particularly comparable. This is of particular interest to be able to examine whether women are exposed to GBD regardless of their career stage. It also allows for direct comparison of any similarities and differences by career stage. In addition, the forms of GBD that occur should be recorded in order to obtain a more differentiated picture of the phenomenon. Previous studies have also addressed this aspect in Germany [28], [32]. However, in these, the forms of sexual harassment were surveyed quantitatively in theory-based closed questions. In contrast, the present study chooses the qualitative method of inductive category formation on answers to an open question. This method seems to be particularly suitable to capture the individual relevance structure of the respondents even in a larger sample.

## Methodology

392 women participated in an online survey on GBD, self-efficacy expectations, and individual perceptions of the “glass ceiling effect” in a medical context in 2019. The survey was conducted as a stand-alone study with funding from the Göttingen Psychological Institute. Female physicians surveyed, were recruited via newsletters from their respective clinics, and female students were recruited via social networks, flyers, and notices in the clinics and universities. All persons who identified with the female gender were addressed. 157 of the respondents were female physicians (*M*_Age_=35.35, SD=8.92), and 235 were female medical students (*M*_Age_=23.95, SD=3.69). Participants came from five university hospitals in northern Germany. In addition to standardized questions on self-efficacy expectations and individual perceptions of the “glass ceiling effect”, the following question was asked to elicit personally experienced GBD: “Have you already had discriminatory experiences at work, in your studies or internship, or the like, because of your gender? If yes, which ones?” The written responses to this question are the subject of this paper. They were analyzed qualitatively according to Mayring’s method of inductive category formation [33]. In this procedure, the statements are summarized step by step in terms of their essential content. This is done in a documented process that includes paraphrasing the statements (i.e., cleaning up redundancy) and several steps of reducing the content to a uniform level of abstraction until finally categories and subcategories are available that condense the respondents’ statements. The content categories extracted from the responses were examined in a second quantifying step to determine how frequently they were mentioned by the female students and physicians surveyed. In accordance with the standard of qualitative research, the responses were coded by a mixed-gender group, and the results were reviewed and interpreted together discursively. In the process, the authors’ own feminist views and their own gender roles were particularly reflected upon.

This combination of qualitative content analysis and quantitative determination of the frequency with which various forms of GBD were mentioned makes it possible to assess the experiences in terms of both content and extent. The chosen method of inductive category formation also offers the advantage of taking into account the individual relevance structure of the respondents, rather than simply obtaining a response to predetermined items. The open-ended question meets the criteria of question design for qualitative online surveys, e.g. clarity and openness. In this case, questions asked online are comparable in quality to interviews [34].

## Results

**Figure 1:**
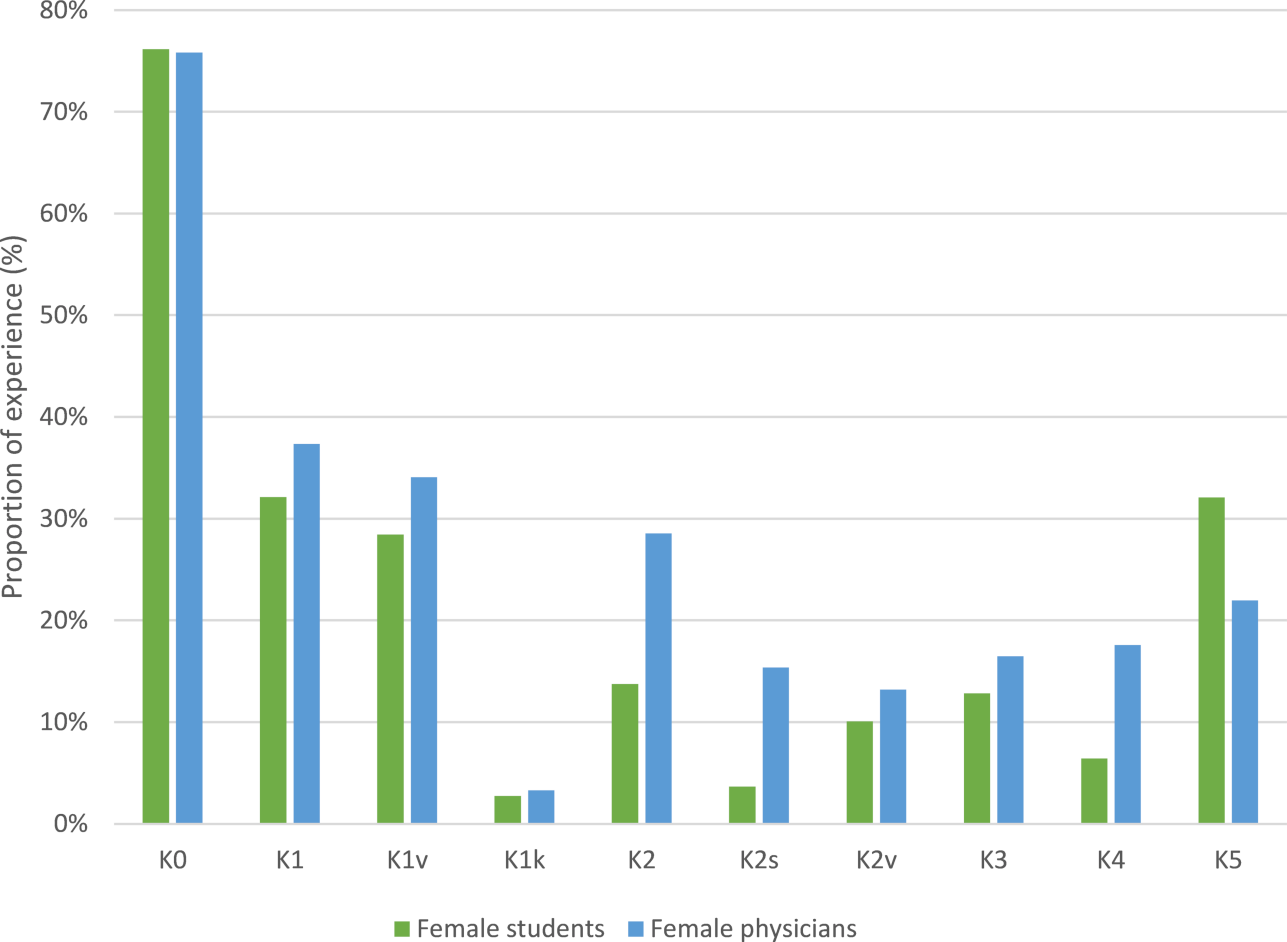
Relative Frequencies of GBD experience per category. Note: N_Students_=109 and N_Physicians_=91. K0=Proportion of women surveyed with GBD experience, K1=Sexual harassment and/or sexist verbal assault, K1v=Verbal harassment, K1k=Physical harassment, K2=Discrimination due to existing/possible motherhood, K2s=Structural discrimination based on existing/possible motherhood, K2v=Verbal discrimination based on existing/possible motherhood, K3=Direct preference for men, K4=Direct neglect of women, K5=Degrading treatment based on gender

46.3% (*n*=109) of female students and 83.5% (*n*=91) of female physicians responded to the question about experiences with GBD. Five main categories were formed from the participants’ statements. Categories 1 and 2 are divided into two subcategories. Structural discrimination in category K2s is to be understood in terms of institutional policies and procedures that lead to inequality between groups such as people of different genders [35]. The relative frequencies of mentions are shown in table 1 (Tab. 1) and figure 1 (Fig. 1). For exemplary responses, see table 2 (Tab. 2). Descriptive differences emerge between female students and female physicians: female students report GBD in the form of K5 more frequently than female physicians; for all other categories, the ratio is reversed.

## Discussion

Around three quarters of female students and physicians report personal experience with GBD. The fact that only 46.3% of the female students and 83.5% of the female physicians answered the open question cannot be clearly interpreted: A non-response should not be understood as a blanket denial; it may also express an unwillingness to share what they have experienced [36]. GBD experiences can be divided into five main categories that can be assigned to different levels of observation, e.g. verbal harassment (K1v; micro level) or structural discrimination based on motherhood (K2s; meso level). The results on sexual harassment (K1) partly correspond to previous findings from German-speaking countries. Among female physicians, the frequency of 37.36% reported here is lower than the 76% reported in the literature [32]. Among female students, the frequency of 32.11% reported here is within the range of values reported in the literature [27], [28], [29], [30], [31]. According to our research, the extent of discrimination based on existing/possible motherhood (K2) was surveyed for the first time in Germany. In an international comparison, the results for female physicians correspond to the frequencies of about one third reported in the USA [37]. With regard to Germany, the category is also evident in qualitative interviews with 20 female physicians about their experiences with discrimination while working in the hospital [38]. The frequent mention of motherhood discrimination (K2) requires additional discussion. Motherhood corresponds to gender conforming behavior. Gender ideology, therefore, cannot be a cause of this form of discrimination. Instead, a hospital’s compulsion for economic success must be considered [39]. Mothers are less available at the workplace, for example because of maternity leave, but also parental leave and care work, which up to now is still rather taken up or borne by mothers than by fathers [40], [41]. Thus, a hospital oriented according to economic interests has the intrinsic motivation not to promote future or current mothers, as they are a less usable labor resource according to economic logic [39]. According to our research, the results on the categories of direct preference for men (K3), direct neglect of women (K4), and degrading treatment based on gender (K5) also cannot be compared with previous literature from Germany, as they were collected for the first time. The categories inductively formed in the present study may be the subject of quantitative hypothesis-testing research in subsequent studies.

Overall, female physicians are affected by all forms of GBD to a greater extent than female medical students, with the exception of degrading treatment based on gender (K5). The present study can only speculate on possible reasons. One conceivable reason is the greater length of time female physicians have spent in the medical system, thus increasing the likelihood of being affected by GBD. However, the specific situation of groups of people in the medical system also differs and may cause different levels of GBD. Future research should shed more light on the differences in the respective situations of female students and female physicians. 

The chosen approach is subject to methodological limitations. On the one hand, trans women and non-binary people were not explicitly addressed during recruitment, although they too may be perceived as female and can thus be affected by GBD. Second, trans men were excluded by the recruitment wording, although they too may be discriminated against for childbearing. Future research should explicitly include these groups of people to paint a more comprehensive picture of GBD. It should be noted that GBD behaviors toward people with queer gender identity take specific forms, and identical behaviors may have different effects on the experience of those affected than for cis women [42]. Furthermore, the results claim validity only for the participants and hospitals studied. However, based on the macro-level causes affecting society as a whole, it seems plausible that the sample studied here does not represent a special case, but that the described extent and forms of GBD also occur in other university and non-university hospitals in Germany. However, this would need to be empirically tested in a survey representative of the whole of Germany. 

Finally, empirically evaluated interventions against GBD will be briefly presented in order to show possible courses of action in practice. Increased awareness for implicit norms can be achieved through explicit education, e.g., through a working group that deals intensively with the topic and monitors developments. An example is the DETECT program of the University of Freiburg [https://www.detect.uni-freiburg.de/]. Such a measure could, for example, have a positive effect on degrading treatment based on gender (K5), as reported here. Another important aspect is the reduction of selection bias, e.g., through a quota of women in departments or committees. This could, for example, have a positive effect on the neglect of women (K4) or preference for men (K3). At the level of organizational structure, isolation of female physicians due to family responsibilities can be reduced by scheduling meetings at family-friendly times or by encouraging interaction in specialty groups [43]. This could address GBD, for example, in the form of structural discrimination based on existing/possible motherhood (K2s). Such interventions have been shown to be effective in reducing GBD [44], [45], [46], [47]. A long-term focus of interventions is important [43], [44] and should target students, physicians, and all groups of people from senior physicians to nurses of any gender [44]. 

Although causes of GBD can be located at all three levels of influence, interventions seem to be mostly proposed at the meso level, where they are also supposed to influence the behavior of perpetrators. The examples given, such as raising awareness for implicit norms of organizational culture [48] or eliminating the isolation of female physicians through inherently discriminatory organizational procedures, illustrate this. The meso level might therefore be a good starting point for interventions because organizational structures are more amenable to change than overall societal gender ideology and patriarchy (macro level) or individual characteristics and beliefs (micro level). See, for example, [45] for small and short-term effects of interventions for the general public.

The results of this study shed light on the high extent and variation of forms of GBD experience for both female medical students and female physicians in the hospitals studied. Furthermore, it seems plausible that the results are transferable to the situation in German hospitals in general. Since interventions are most likely to succeed at the meso level, it is important to emphasize the responsibility of employers and medical educators to recognize and address GBD as a prevalent and complex problem. Numerous findings on causes and consequences as well as empirically evaluated interventions are available for this task, which should be specifically selected and implemented in hospitals.

## Data

Data for this article are availabe from Dryad Repository: [https://doi.org/10.5061/dryad.1rn8pk0xs] [49]

## Acknowledgements

We acknowledge support by the Open Access Publication Funds of the Göttingen University. The German version of this manuscript was translated into English with the help of DeepL. The translation was proofread by Paul J. Pritz.

## Competing interests

The authors declare that they have no competing interests. 

## Figures and Tables

**Table 1:**
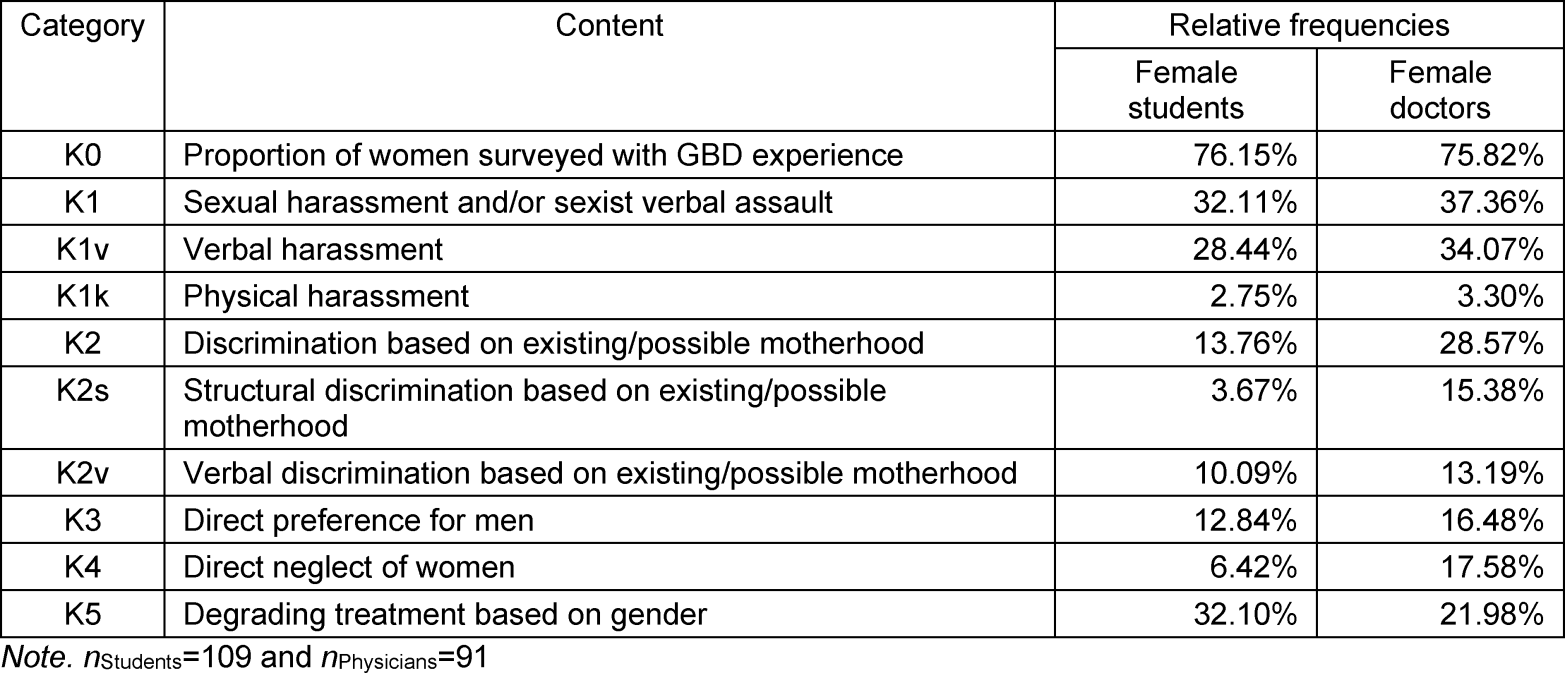
Relative frequencies of GBD experience per category

**Table 2:**
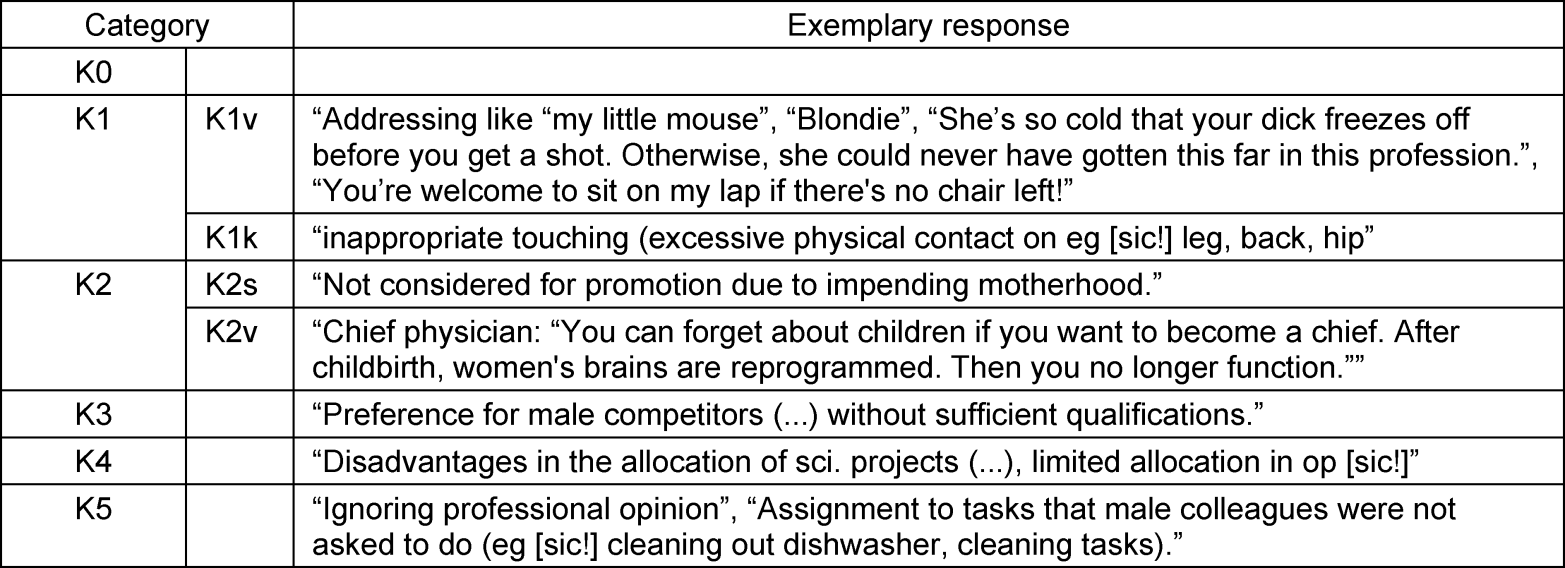
Exemplary responses per category
